# The Lipoteichoic Acid-Related Proteins YqgS and LafA Contribute to the Resistance of Listeria monocytogenes to Nisin

**DOI:** 10.1128/spectrum.02095-21

**Published:** 2022-02-23

**Authors:** Xinxin Pang, Yansha Wu, Xiayu Liu, Yajing Wu, Qin Shu, Jianrui Niu, Qihe Chen, Xinglin Zhang

**Affiliations:** a Department of Food Science and Nutrition, Zhejiang University, Hangzhou, China; b College of Agriculture and Forestry, Linyi University, Linyi, China; Universidad de Buenos Aires, Facultad de Farmacia y Bioquimica

**Keywords:** *Listeria monocytogenes*, transposon sequencing (Tn-seq), biofilm, lipoteichoic acid, nisin, pathogenicity

## Abstract

Listeria monocytogenes is a major pathogen contributing to foodborne outbreaks with high mortality. Nisin, a natural antimicrobial, has been widely used as a food preservative. However, the mechanisms of L. monocytogenes involved in nisin resistance have not yet to be fully defined. A *mariner* transposon library was constructed in L. monocytogenes, leading to the identification of 99 genes associated with the innate resistance to nisin via Transposon sequencing (Tn-seq) analysis. To validate the accuracy of the Tn-seq results, we constructed five mutants (Δ*yqgS*, Δ*lafA*, Δ*virR*, Δ*gtcA*, and Δ*lmo1464*) in L. monocytogenes. The results revealed that *yqgS* and *lafA*, the lipoteichoic acid-related genes, were essential for resistance to nisin, while the *gtcA* and *lmo1464* mutants showed substantially enhanced nisin resistance. Densely wrinkled, collapsed surface and membrane breakdown were shown on Δ*yqgS* and Δ*lafA* mutants under nisin treatment. Deletion of *yqgS* and *lafA* altered the surface charge, and decreased the resistance to general stress conditions and cell envelope-acting antimicrobials. Furthermore, YqgS and LafA are required for biofilm formation and cell invasion of L. monocytogenes. Collectively, these results reveal novel mechanisms of nisin resistance in L. monocytogenes and may provide unique targets for the development of food-grade inhibitors for nisin-resistant foodborne pathogens.

**IMPORTANCE** Listeria monocytogenes is an opportunistic Gram-positive pathogen responsible for listeriosis, and is widely present in a variety of foods including ready-to-eat foods, meat, and dairy products. Nisin is the only licensed lantibiotic by the FDA for use as a food-grade inhibitor in over 50 countries. A prior study suggests that L. monocytogenes are more resistant than other Gram-positive pathogens in nisin-mediated bactericidal effects. However, the mechanisms of L. monocytogenes involved in nisin resistance have not yet to be fully defined. Here, we used a mariner transposon library to identify nisin-resistance-related genes on a genome-wide scale via transposon sequencing. We found, for the first time, that YqgS and LafA (Lipoteichoic acid-related proteins) are required for resistance to nisin. Subsequently, we investigated the roles of YqgS and LafA in L. monocytogenes stress resistance, antimicrobial resistance, biofilm formation, and virulence in mammalian cells.

## INTRODUCTION

Listeria monocytogenes is an intracellular foodborne pathogen, leading to a severe invasive infection called listeriosis after consumption of contaminated food (1, 2). Listeriosis is a deadly infection resulting in poor survival rates among all foodborne diseases, with up to 20% to 30% mortality (3). L. monocytogenes is ubiquitous in the environment, which is also widely present in a variety of foods including ready-to-eat foods, meat, and dairy products (4–6). Given its ability to adapt to adverse environmental conditions (such as high salt, low temperatures, and pH), the contamination of ready-to-eat foods is particularly concerning (7, 8). Therefore, controlling the survival and reproduction of L. monocytogenes in ready-to-eat foods is a thorny problem, which needs to be urgently addressed.

Bacteriocins are antimicrobial peptides with broad-spectrum antibacterial activities produced by bacteria, which have been used as preservatives to control pathogen propagation in foods for many decades (9, 10). Nisin is the only licensed lantibiotic by the FDA for use as a food grade inhibitor in over 50 countries (11, 12). Generally, nisin, as a cationic antimicrobial peptide, binds to the bacterial cell surface and oligomerizes in cytoplasmic membranes to form pores, leading to the leakage of cellular contents and depolarization of the membrane potential, and eventually cell death (13). In addition, nisin inhibits cell wall synthesis through interacting with lipid II (14). This dual mechanism of action plays a synergistic role in killing bacteria (15).

The intrinsic resistance to nisin of L. monocytogenes was first described in 1991 (16). Remarkably, a prior study suggests that L. monocytogenes is more resistant than other Gram-positive pathogens in nisin-mediated bactericidal effects (17). Despite the mechanisms of nisin have been identified, the association of genes with nisin resistance in L. monocytogenes is not fully understood. Several loci have been identified in L. monocytogenes that can lead to nisin resistance, including the two-component systems LisRK (18), VirRS (19), and LiaR (20), the alternative stress sigma factor SigB (21), the penicillin-binding protein Pbp4 (22), the class three stress gene regulator CtsR (23), the glutamate decarboxylase GadD1 (24), the ABC transporters AnrAB (25) and VirAB (26), lysinylation of phospholipids MprF (27), d-alanyl-teichoic acids biosynthesis DltA (28), and the tellurite resistance protein TelA (29). Although previous studies have reported many genes associated with nisin resistance, a number of nisin-related genes are not yet fully found. With the improvement of sequencing technology, transposon sequencing (Tn-seq), as an efficient microbial functional genomic tool, is widely used to study the association between phenotypes and genotypes in pathogens (30). Compared with conventional mutation approaches, Tn-seq has the advantage of high efficiency, accuracy, and wide coverage (31, 32).

Lipoteichoic acid (LTA), as an important bacterial cell wall component, is a zwitterionic polymer and is typically composed of polyglycerolphosphate backbone chain tethered to the membrane by a glycolipid (Gal-Glc-DAG or Gal-Ptd-6Glc-DAG) (33). In L. monocytogenes, the glycosyltransferases LafA (Lmo2555) and LafB (Lmo2554) are required for the formation of Glc-DAG and Gal-Glc-DAG, respectively(34). YqgS (Lmo0644) and LtaS (Lmo0927) are involved in polyglycerolphosphate backbone synthesis, YqgS transfers the initial glycerolphosphate onto the glycolipid anchor and LtaS extends the glycerolphosphate backbone chain(34). LTA mutants are defective in biofilm formation and reduced virulence in Gram-positive bacteria, such as Staphylococcus aureus, Enterococcus faecalis, and Bacillus subtilis (35–39).

In this study, we used Tn-seq to identify genes involved in nisin resistance via a high-density transposon library of L. monocytogenes EGD-e. In addition to the nisin-resistance-associated genes validated previously, a large amount of novel genes with great significance were identified. YqgS and LafA are involved in the LTA backbone and glycolipid linked to the membrane, respectively (34). Moreover, there has been no systematic study into the relationship between these genes and nisin resistance. Further investigations of Δ*yqgS* and Δ*lafA* mutants revealed an associated enhanced surface negative charge and reduced capacity to form biofilm. In addition to revealing the function of YqgS and LafA in various antibiotics and stress resistance, we have also explored the role of LTA in *Listeria* pathogenicity.

## RESULTS

### Illustration of the experimental protocols of Tn-seq.

**FIG 1:**
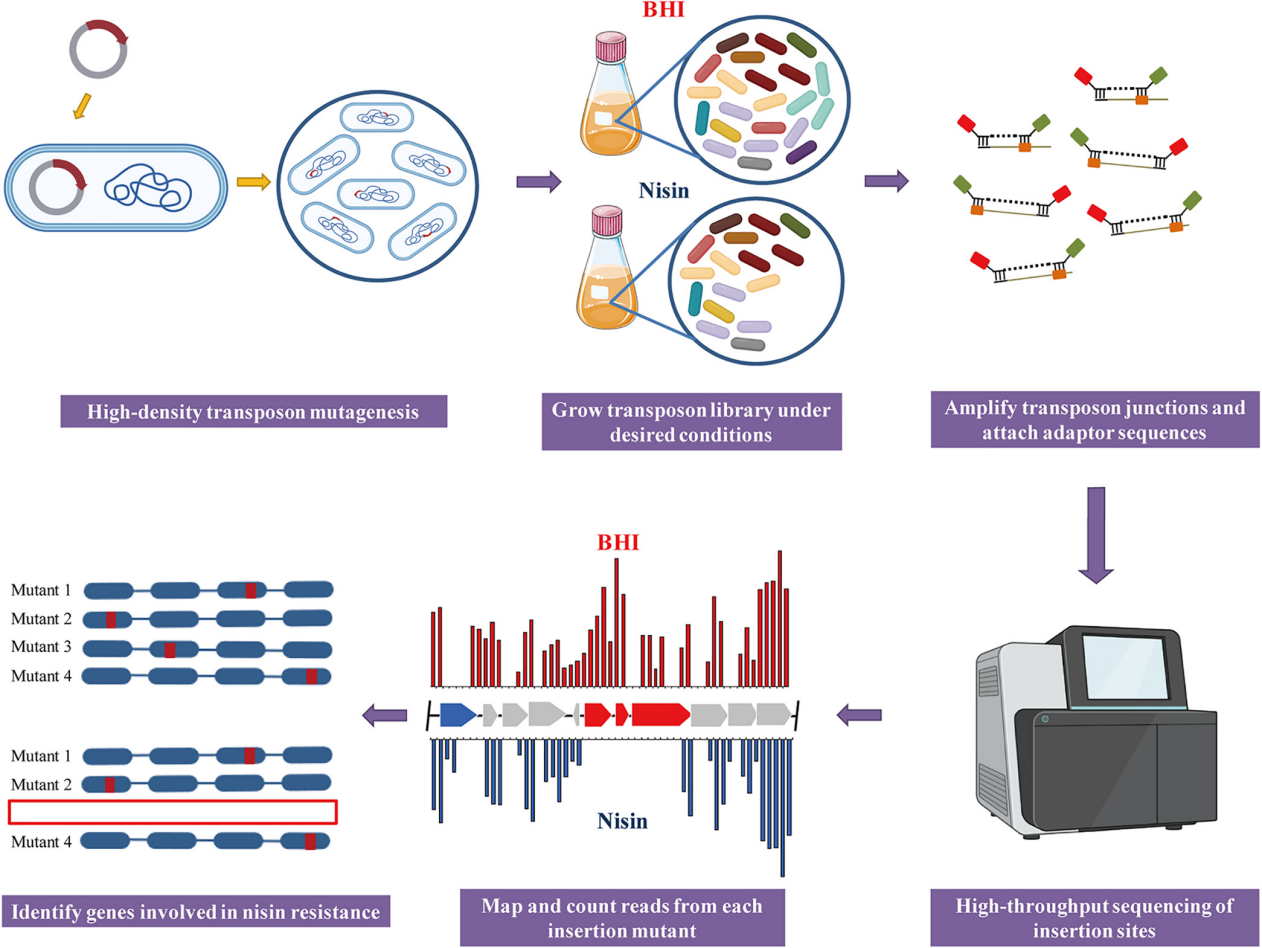
Schematic illustration of the Tn-seq. First, a high-density transposon mutant library was constructed and cultured in different conditions. Genomic DNA was extracted. Then, sequencing adaptors and barcodes are added to DNA fragments, which could be amplified by using PCR. Subsequently, samples were analyzed by sequencing to map and count reads from each mutant. Finally, genes involved in nisin resistance were identified.

To identify genome-wide genes associated with nisin resistance, a *mariner*-based transposon insertion library was generated in L. monocytogenes EGDe. The main steps of Tn-seq are illustrated in Fig. 1. In brief, Tn-seq was performed on cultures of the L. monocytogenes transposon mutant library. This library was grown in brain heart infusion (BHI) with or without subinhibitory concentration of nisin (200 μg/mL). Subsequently, DNA was extracted from samples, and we amplified transposon junctions and attached adaptor sequences to construct a DNA library for sequencing. The sequencing results indicated that each sample had at least 10 million Tn-seq readings and no biased insertion sites of the transposon were identified. The distribution of transposon was in an even and high-density pattern, and 43,793 unique insertion sites were identified throughout the genome (Supplementary File 2). Finally, analysis of Tn-seq results was carried out to identify genes associated with nisin resistance.

### Identification of genes associated with nisin resistance by Tn-seq.

**FIG 2:**
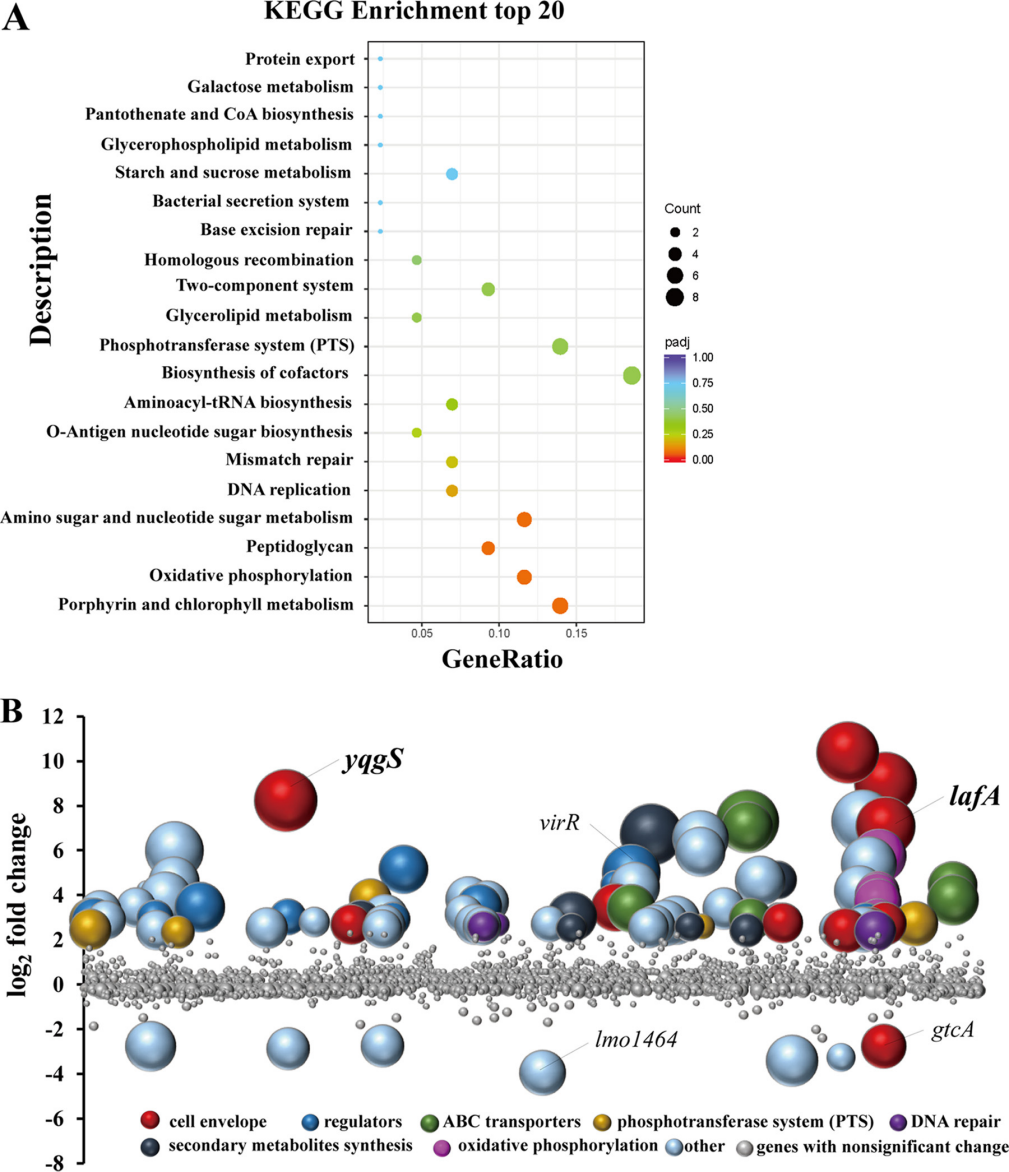
Identification of genes involved in nisin resistance by Tn-seq analysis. (A) Scatterplot of KEGG enrichment analysis. The enriched biological pathways are shown on the *y* axis; the ratio of differential genes to the total genes is indicated on the *x* axis. (B) Tn-seq analysis to identify genes associated with nisin resistance in L. monocytogenes. The bubbles represent genes, the size of the bubble represents the significance of genes with -log_10_ (BH value) (fold > 5, BH < 0.05). The abscissa represents the position of a gene on the genome and the ordinate indicates the log_2_ (fold changes) derived from the read-count ratio of libraries grown in BHI to libraries grown in BHI with nisin. Colors represent gene functional categories. All the genes with nonsignificance were marked in gray, which BH were limited to be no more than 0.7 in this figure for a clearer view.

We identified 99 genes, and the number of transposon insertions was significantly different among groups in the presence or absence of nisin (Benjamini-Hochberg < 0.05), which were involved in nisin resistance (Fig. 2A and B). Ninety-two of them were inactivated by transposon insertion, leading to increased nisin sensitivity in the corresponding mutant (Table S2). Namely, the expression products of disrupted genes, some of which were clustered into several particular functional categories (Fig. 2B), were favorable for the resistance of nisin. Eleven genes are related to the cell envelope, which encodes cell-division protein (*ftsW*), peptidoglycan lytic protein P45 (*spl*), glycolipid and polyglycerolphosphate lipoteichoic acid synthesis (*yqgS* and *lafABC*), d-alanyl-teichoic acids biosynthesis (*dltA*), lysinylation of phospholipids (*mprF*), penicillin-binding protein (*pbp4*), d-alanyl-alanine synthetase (*ddl*), and UDP-glucose 4-epimerase (*galE*). Ten regulators genes were identified, including seven putative transcriptional regulators (*lacI* family, *ctsR* family, *gntR* family, *marR* family, *arsR* family, *lmo0651*, and *lmo1262*), two 2-component response regulators (*liaR* and *virR*), and a recombination regulator (*recX*).

The ABC transporters are known to exert important functions in antimicrobials resistance associated with efflux systems (40–42). Out of the seven newly identified ABC transporter genes, three genes (*virA* and *anrAB*) were useful to confer nisin resistance in L. monocytogenes, which were regulated via two-component systems (26). Phosphotransferase system (PTS) regulates carbohydrate metabolism by catalyzing function in sugar transport and phosphorylation (43), mannose PTS permease, and β-glucoside PTS enzyme II were involved in sensitivity to class IIa bacteriocins (44–46). Six putative PTS transports genes (*lmo0021*, *lmo0024*, *lmo0301*, *lmo0916*, *lmo1971*, and *lmo2649*) were identified. The DNA repair genes are essential for DNA damage caused by antimicrobial treatment (47), of which three genes with significant fold decrease in transposon insertions include DNA polymerase (*polC*), NAD-dependent DNA ligase (*ligA*), and single-strand DNA-binding protein (*lmo2523*). Moreover, eight genes involved in the biosynthesis of secondary metabolites were identified, six genes (*hemA*, *hemC*, *henE*, *hemH*, *lmo2113*, and *lmo0884*) of which were clustered in the porphyrin and chlorophyll metabolism pathway. And five susceptibility genes are located in the *atpABCDEFG* operon (*atpA*, *atpB*, *atpD*, *atpG*, *atpH*), which is involved in membrane bioenergetics such as ATP synthase (48), and previous studies suggest that ATP synthesis is related to cell attachment and biofilm stability (49, 50).

Seven genes were identified as contributing to nisin sensitivity (Table S3). That is, the corresponding transposon mutants were significantly enriched in the presence of nisin compared with the untreated group. Of those, two genes (*lmo1464* and *gtcA*) were linked to cell envelope, which encoded diacylglycerol kinase and wall teichoic acid glycosylation protein GtcA, respectively. The other five genes with significant fold decrease are annotated as monovalent cation/H+ antiporter subunit C (*lmo2380*) and hypothetical proteins (*lmo2258*, *lmo0955*, *lmo0653*, *lmo0215*). Overall, the transposon library screening has comprehensively identified genes that confer resistance or are important for the mechanisms of action of nisin at the genome-wide scale.

### Lipoteichoic acid is essential for L. monocytogenes resistance to nisin but not to bacitracin.

**FIG 3:**
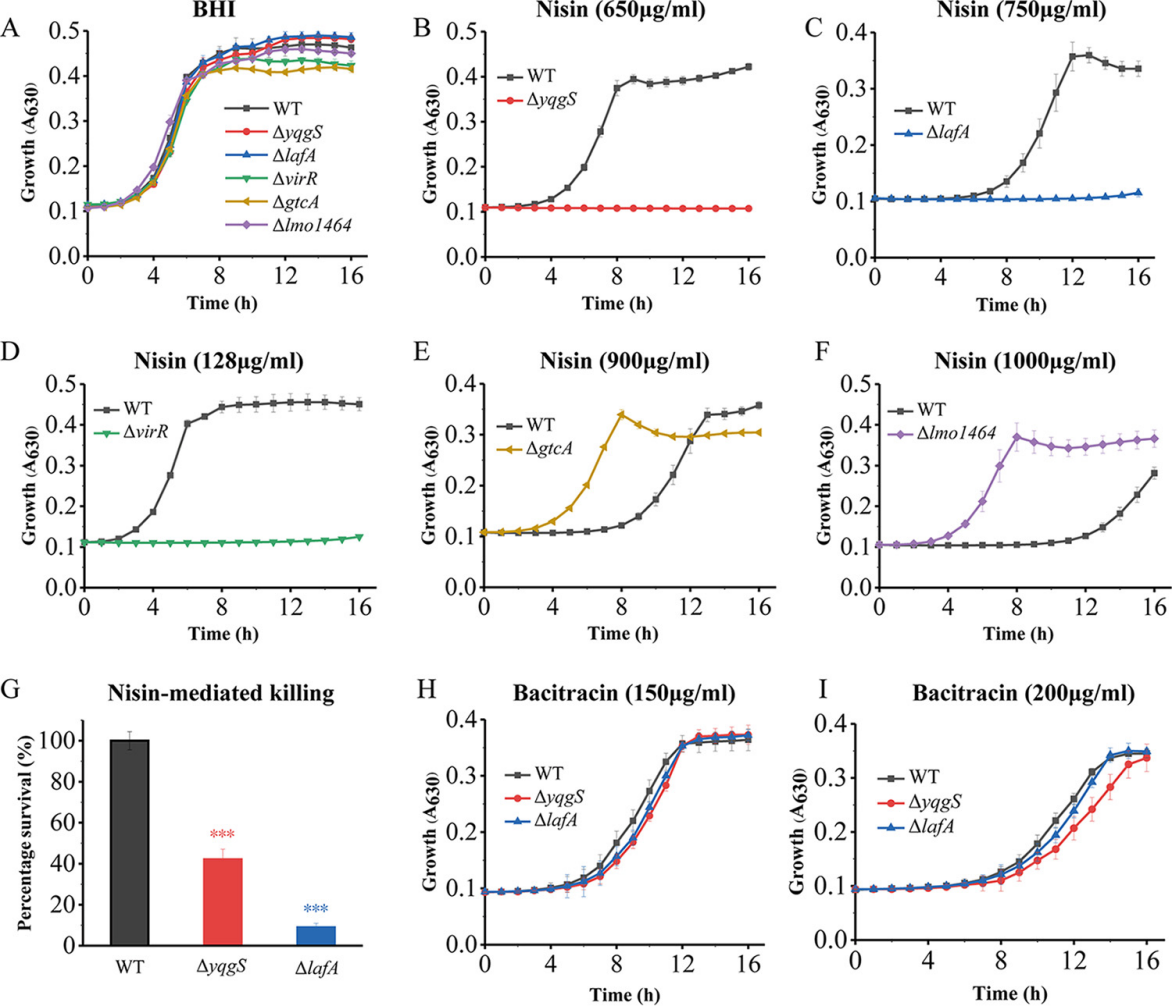
YqgS and LafA are required for nisin resistance but not for bacitracin. (A to F) Growth curves of strain EGDe and different mutants in the absence (A) or presence of nisin with 650 μg/mL (B), 750 μg/mL (C), 128 μg/mL (D), 900 μg/mL (E), and 1,000 μg/mL (F). (G) Survival percentage of L. monocytogenes strains following a nisin challenge (1,000 μg/mL). (H, I) Growth of L. monocytogenes strains in BHI supplemented with 150 μg/mL (H) or 200 μg/mL (I) bacitracin. Data represent mean of three independent experiments. ***, *P* ≤ 0.001.

In order to validate the Tn-seq results, we constructed five mutants (Δ*yqgS*, Δ*lafA*, Δ*virR*, Δ*gtcA*, and Δ*lmo1464*), which were identified with high fold changes and significant *P*-values. The VirRS signal transduction system is known to determine the nisin resistance, yet, whether the deficiency in the other four genes changes nisin sensitivity has not been investigated. Under nisin-free conditions, the growth rate or cell density in the stationary phase of mutants was not significantly changed compared with the wild-type strain (Fig. 3A). The growth of *yqgS* mutant was grossly affected in the presence of nisin (650 μg/mL), and nisin (750 μg/mL) also had a severe effect on the growth of *lafA* mutant (Fig. 3B and C). In contrast, inactivation of the *virR* gene has the strongest negative impact on nisin resistance (Fig. 3D). The *gtcA* and *lmo1464* mutants showed significantly enhanced resistance to nisin (Fig. 3E and F). The results of the validation experiment were also consistent with the previous Tn-seq results. In addition, deletion of *yqgS* and *lafA* indeed increased L. monocytogenes susceptibility to nisin-mediated killing (Fig. 3G). Next, we determined the growth in mutant and wild-type strains under bacitracin conditions, which also blocks cell wall synthesis by binding to the lipid carrier of peptidoglycan subunits as nisin (51, 52). The growth speed of *yqgS* and *lafA* mutants revealed a slight reduction compared with wild-type, although this was not statistically significant (Fig. 3H and I). These genes play a role in glycolipid (*lafA*) and lipoteichoic acid backbone (*yqgS*) synthesis, indicating that Lipoteichoic acid is essential for nisin resistance but not to bacitracin in L. monocytogenes.

### Examination of morphology.

**FIG 4:**
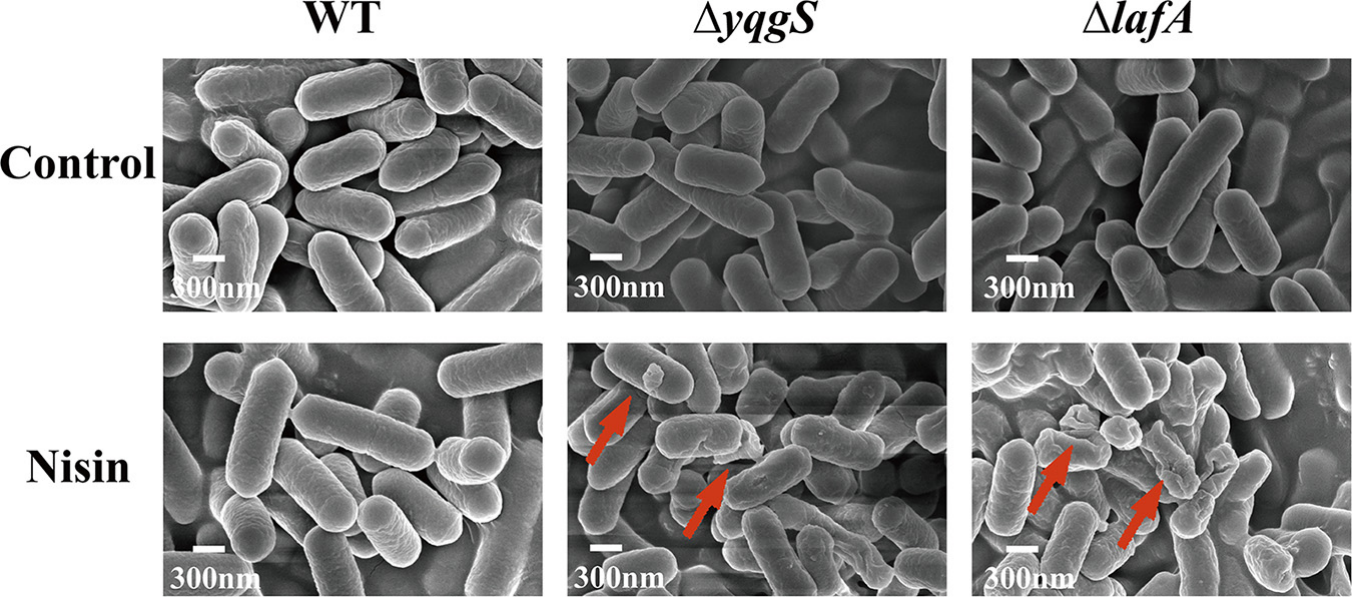
The SEM micrograph of L. monocytogenes strains untreated or treated with nisin (800 μg/mL). Arrows indicate the damage of nisin treated L. monocytogenes.

**FIG 5:**
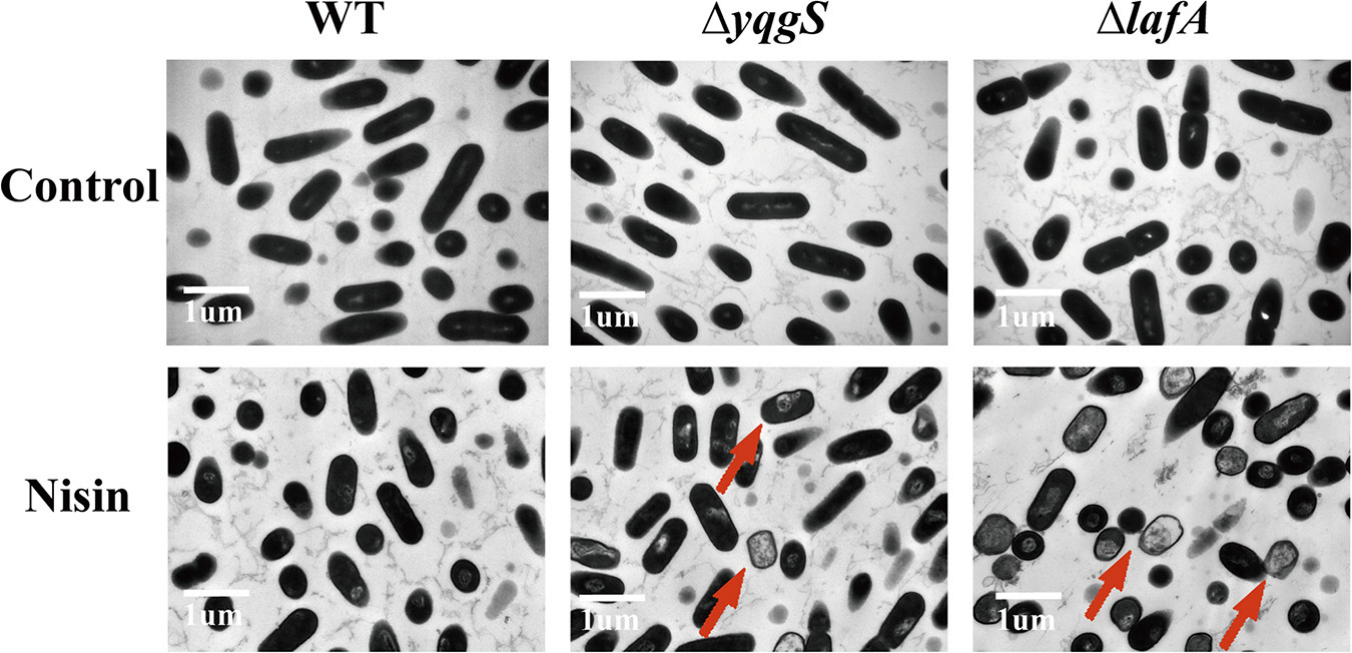
The TEM micrograph of L. monocytogenes strains untreated or treated with nisin (800 μg/mL). Arrows indicate the damage of nisin treated L. monocytogenes.

To further explore the effect of nisin on the wild-type and mutant strains, we performed scanning electron microscopy (SEM) and transmission electron microscopy (TEM) scans of the nisin-treated bacterial cultures. No significant difference is observed between the wild-type and mutant strains as displayed in Fig. 4 and 5 (control). After 1-h exposure to nisin (800 μg/mL), wild-type strain showed no visible alteration, but Δ*yqgS* and Δ*lafA* mutants exhibited striking changes in cell morphology, including densely wrinkled surface, collapsed surface, membrane breakdown, and cellular content discharge from the cell at a breach point (Fig. 4 and 5). Overall, these results suggested that the Δ*yqgS* and Δ*lafA* mutants are more susceptible to nisin-mediated bactericidal action than wild-type.

### YqgS and LafA affect the surface charge of L. monocytogenes.

**FIG 6:**
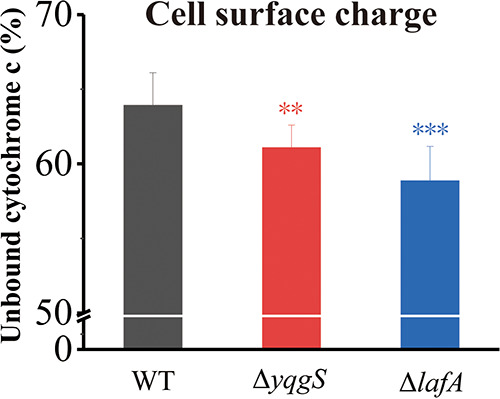
Cell surface charge analysis of L. monocytogenes strains. Data represent the mean ± SD of three independent experiments. **, *P* ≤ 0.01; ***, *P* ≤ 0.001.

To determine if the change of surface charge resulted in the reduced amount of nisin adsorbed to the surface of Δ*yqgS* and Δ*lafA* mutants, the surface charge of the mutant and wild-type strains was determined by measuring the binding of cytochrome c. As expected, we observed higher levels of cytochrome c binding with the Δ*yqgS* and Δ*lafA* mutants than wild-type strain (Fig. 6), indicating that the absence of glycolipid in LTA slightly enhanced the surface negative charge, and the absence of LTA, to a greater extent, increased the negative charge on the bacterial surface. Throughout, the result allowed us to discard electrostatic changes at the bacterial surface as a reason for increased nisin sensitivity on the mutants.

### Influence of YqgS and LafA on biofilm formation in L. monocytogenes.

**FIG 7:**
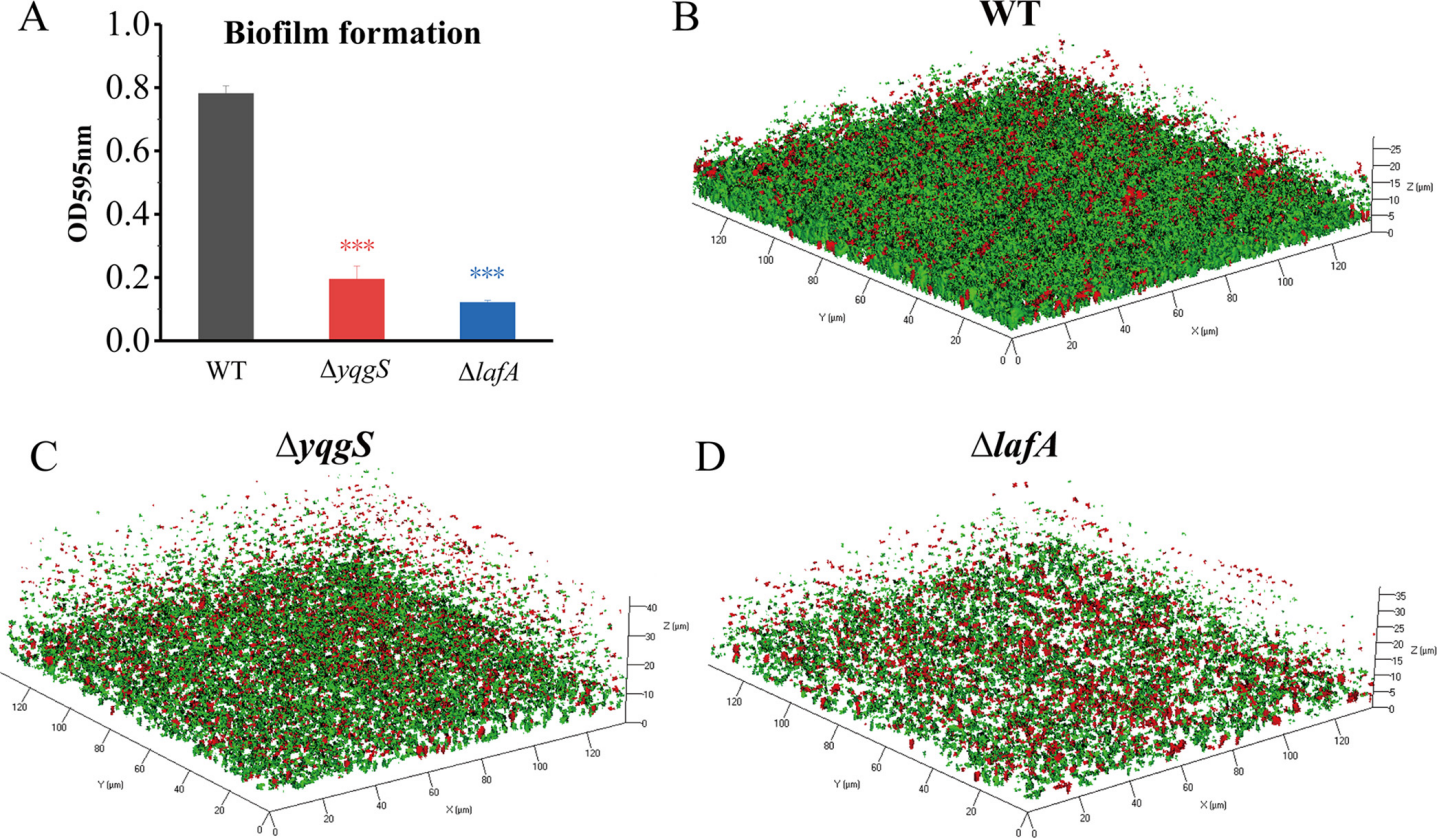
YqgS and LafA regulate biofilm formation. (A) Biofilm formation of wild-type, Δ*yqgS*, and Δ*lafA* mutant strains, which was measured by the crystal violet assay. Confocal microscopy images of wild-type (B), Δ*yqgS* (C), and Δ*lafA* (D) biofilm. Green color cells represent viable cells with intact membranes and red color cells are dead cells. The experiments were performed in triplicate. ***, *P* ≤ 0.001.

Previous studies showed that the lipoteichoic acid mutation of S. aureus resulted in complete loss of the biofilm-formation capacity on plastic (35). However, the capacity of the L. monocytogenes LTA mutant strain to form biofilm has not been investigated. To inquire whether YqgS and LafA are associated with the formation of biofilms, crystal violet assay was performed (53). We observed that Δ*yqgS* and Δ*lafA* mutants displayed a greatly reduced capacity to form biofilm relative to wild-type strain (Fig. 7A). Subsequently, we also investigated the role of YqgS and LafA in biofilm formation by confocal laser scanning microscope (54). Three-dimensional analysis of 2-day-old biofilms confirmed that the *yqgS* and *lafA* mutants formed a less dense and relatively thin biofilm compared with wild-type (Fig. 7B to D). Altogether, these results demonstrated that YqgS and LafA are required for biofilm formation.

### Impact of YqgS and LafA on antibiotic sensitivity and stress resistance.

To study the role of YqgS and LafA as LTA-related proteins in antibiotic resistance, we assessed the antimicrobial sensitivity of Δ*yqgS* and Δ*lafA* mutants by antibiotic disk agar-based assays. The assay results demonstrated that genetic inactivation of YqgS and LafA results in markedly increased antibiotics susceptibility, including oxacillin, polymixinB, cefuroxime, ceftriaxone, and carbenicillin (Table 1). An inactivating mutation at the *yqgS* gene can also increase penicillin sensitivity but no significant changes in resistance to ampicillin, cefotaxime, ciprofloxacin, amikacin, erythromycin, and chloramphenicol. In addition, significant decreases in cefotaxime resistance were detected in Δ*lafA* mutant. Meanwhile, mutations in the LTA system were linked to increased resistance to vancomycin and gentamicin. It is thus apparent that YqgS and LafA greatly contribute to the resistance of L. monocytogenes to various important cell envelope-targeting antimicrobials by affecting bacterial cell envelope integrity.

**TABLE 1:** Relative sensitivities of mutants and wild-type strains to a variety of antibiotics

Antibiotic	Mean size of the zone of inhibition (mm) ± SD^*a*^		
	EGD-e	Δ*yqgS* mutant	Δ*lafA* mutant
Penicillin	34.00 ± 0.72	37.50 ± 0.50**	34.50 ± 0.50
Ampicillin	30.93 ± 0.12	31.03 ± 1.00	31.67 ± 0.58
Vancomycin	20.13 ± 0.15	18.83 ± 0.29**	18.10 ± 0.10***
Oxacillin	9.83 ± 0.47	12.13 ± 0.35**	11.90 ± 0.26**
PolymixinB	7.00 ± 0.00	9.80 ± 0.70**	10.83 ± 0.35***
Cefuroxime	21.00 ± 0.20	25.50 ± 0.50***	30.67 ± 0.58***
Ceftriaxone	20.56 ± 0.25	21.27 ± 0.25*	26.03 ± 0.06***
Carbenicillin	33.93 ± 0.12	36.50 ± 0.50***	35.40 ± 0.35**
Cefotaxime	20.63 ± 0.15	21.10 ± 0.36	24.73 ± 0.64***
Gentamycin	24.83 ± 0.47	23.10 ± 0.36**	22.83 ± 0.35**
Ciprofloxacin	23.17 ± 0.29	23.03 ± 0.06	23.43 ± 0.40
Amikacin	21.93 ± 0.12	21.80 ± 0.82	21.80 ± 0.26
Erythromycin	28.27 ± 0.25	27.80 ± 0.44	28.03 ± 0.25
Clindamycin	19.77 ± 0.68	17.93 ± 0.12**	18.57 ± 0.51
Chloramphenicol	26.777 ± 0.40	26.60 ± 0.36	24.83 ± 0.15**

a Results are mean ± SD of three independent experiments.*, *P* ≤ 0.05; **, *P* ≤ 0.01; ***, *P* ≤ 0.001.

**FIG 8:**
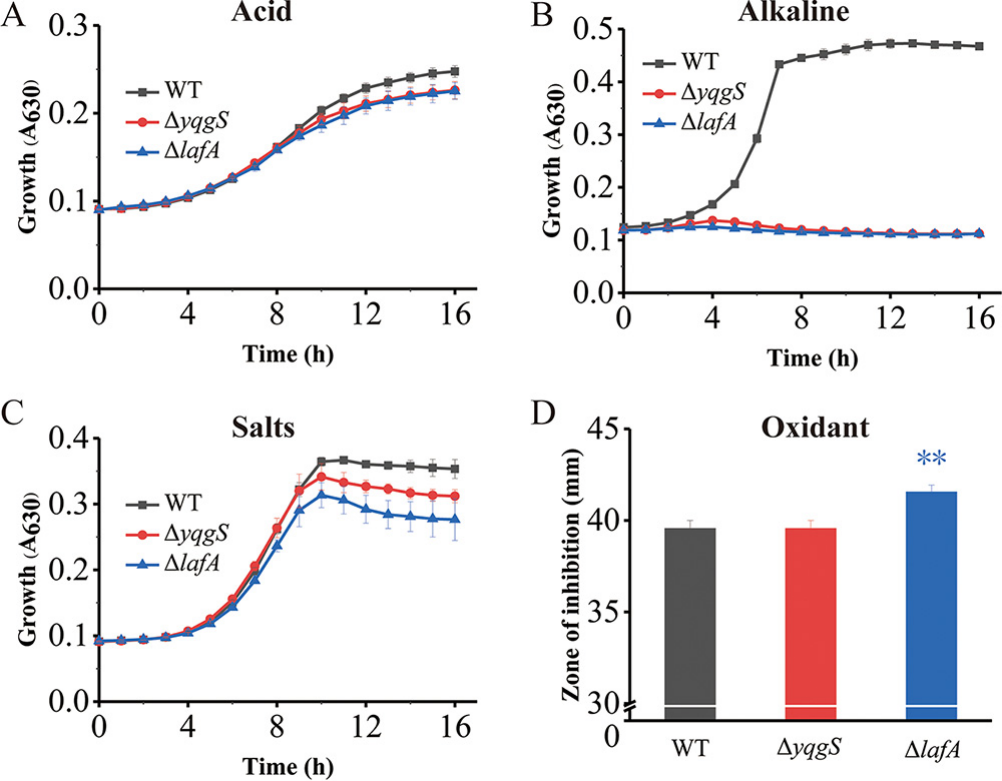
Impact of *yqgS* and *lafA* deletion on growth under unfavorable conditions. The growth curves of L. monocytogenes wild-type, Δ*yqgS*, and Δ*lafA* mutants in unfavorable conditions, including pH 6.0 (A), pH 9.5 (B), 750 mM NaCl (C). (D) The size of the inhibition zone of the WT and mutants with 30% H_2_O_2_ (15 μL).

To evaluate the function of YqgS and LafA on stress resistance, we further assessed the effects of environmental pressure from food processing (acid, alkaline, salts, and oxidant) on bacterial growth in different strains. The resistance of Δ*yqgS* and Δ*lafA* mutants to acid (Fig. 8A) and salts (Fig. 8C) was slightly decreased compared with wild-type strain. The growth of mutants was severely affected under alkaline conditions (Fig. 8B). These results above suggest that the destruction of cell wall-related gene functions may lead to a drastic effect on coping pH change for L. monocytogenes, and indicate that pH is a critical factor for enhanced bacteria killing in particular circumstances. In addition, the antioxidant capacity of Δ*lafA* mutant was considerably attenuated (Fig. 8D). Overall, YqgS and LafA are dispensable for general stress resistance in L. monocytogenes.

### YqgS and LafA are required for intracellular multiplication.

**FIG 9:**
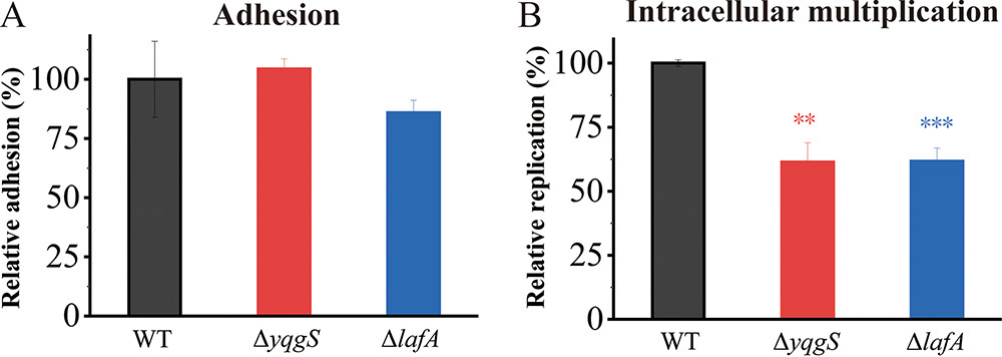
RAW264.7 macrophages were infected with wild-type, Δ*yqgS*, and Δ*lafA* mutant strains. (A) Adhesion of RAW264.7 by different strains as CFU counts relative to wild-type (fixed at 100%). (B) Intracellular multiplication behavior of mutants and wild-type strains in RAW264.7. Results are mean ± SD of three independent experiments. **, *P* ≤ 0.01; ***, *P* ≤ 0.001.

Given the fact that YqgS and LafA increase the surface negative charge and play an important role in biofilm formation, we hypothesized that the LTA-related genes are essential for L. monocytogenes pathogenicity. To test this assumption, we infected the RAW264.7 murine macrophage-like cells with different strains (wild-type, Δ*yqgS*, and Δ*lafA* mutant) and determined the number of intracellular bacteria as a function of time. No obvious differences were detected in adherence from all the strains (Fig. 9A), but the multiplication rates of the mutants exhibited partial defects upon internalization in the RAW (Fig. 9B). In sum, these results showed that deletion of *yqgS* and *lafA* had no effect on adhesion, but these are crucial in efficient survival and replication of L. monocytogenes in macrophages.

## DISCUSSION

In this study, 99 genes associated with nisin resistance have been identified in L. monocytogenes at a genome-wide scale via Tn-seq. Of these, 92 genes contribute to nisin resistance, and only seven genes exert negative roles on nisin resistance. Previous studies have demonstrated that the cell envelope-related genes of *dltA*, *mprF*, and *lmo2229* contributed to the resistance of L. monocytogenes to nisin (22, 27, 28, 55). The DltA mediates the d-alanylation of teichoic acids and the MprF is required for lysinylation of phospholipids, which reduce the surface negative charge of bacterial cells, decrease the binding of CAMPs, and consequently enhance resistance to nisin (27, 56–59). The *pbp4* mutant showed increased nisin sensitivity likely via regulation of peptidoglycan peptide cross-links (22, 55). The changes of the cytoplasmic membrane, such as the presence of less diphosphatidylglycerol and more phosphatidylethanolamine, were generally related to the impaired nisin effectiveness (60, 61). Therefore, in this study we focused on two cell envelope-related genes, which were involved in glycolipid (*lafA*) and lipoteichoic acid backbone (*yqgS*) synthesis.

Resistance to nisin is attributed to stress response mechanisms, and it has been reported previously that two-component systems (*lisRK*, *liaRS*, and *virRS*) perform important functions in innate lantibiotic resistance (18, 26, 62–64). Strikingly, out of the 27 genes regulated by *liaRS* (65), seven genes (*lmo0954*, *virA*, *lmo1966*, *telA*, *pbp4*, *lmo2484*, and *lmo2485*) were identified as nisin-resistance genes, the three resistance genes of which were previously described including *virA* (26, 64), *telA* (29), and *pbp4* (22), encoding ABC transporter permease, toxic ion resistance protein, and penicillin-binding protein, respectively. In addition, five VirR-regulated genes (*anrAB*, *dltA*, *mprF*, and *lmo2156*) were correlated with resistance against nisin, which also were verified experimentally in previous studies (27, 28, 64) except for the gene of *lmo2156*.

The reidentification of locus previously shown to be related to nisin resistance confirms the reliability of the screening, but two known nisin-resistance genes (*lisK* and *gadD1*) have not been identified. The signal transduction system LisK and the glutamate decarboxylase GadD1 determined the sensitivity of L. monocytogenes LO28 to nisin (18), but the corresponding genes were not identified in L. monocytogenes EGDe, presumably due to the genetic difference of strains or variation in experimental conditions (66). In addition, the newly identified genes (*yqgS*, *lafA*, *gtcA*, and *lmo1464*) were deleted to validate the accuracy of Tn-seq analysis. We have demonstrated the role of GtcA and Lmo1464 in nisin sensitivity, and these mutants showed significantly enhanced resistance to nisin. However, the detailed mechanism still needs to be investigated.

LTAs are important cell wall polymers usually composed of polyglycerolphosphate backbone chain that is embedded in the membrane via a glycolipid, which plays critical roles in the maintenance of antimicrobial resistance and pathogenicity of Gram-positive pathogens (39, 67). LafABC, involved in glycolipid synthesis, are required for LTA synthesis in L. monocytogenes. The LTA primase YqgS (also called LtaP) and synthase LtaS (Lmo0927) are responsible for LTA backbone synthesis (34). We identified all the LTA-related genes in our Tn-seq analysis except for *ltaS*, which is potentially attributable to the severe growth defect of *ltaS* mutant (34). LTA-related genes (*yqgS* and *lafA*) are essential for L. monocytogenes resistance to nisin but not to bacitracin. LTA was previously reported to be involved in nisin resistance of Streptococcus bovis and S. aureus (68, 69).

Generally, nisin binds to the bacterial cell surface and oligomerizes in cytoplasmic membranes to form pores, leading to the leakage of cellular contents and cell death (13). The binding affinity of nisin toward the surface of the bacteria is driven by electrostatic forces between cationic peptides and negatively charged cell envelope (70), and modification of LTAs is known to accumulate positive charge on the bacterial surface to decrease the effectiveness of nisin (28, 71). The increased negative charge on the surface of the *yqgS* and *lafA* mutants may be part of the reason for the increased nisin sensitivity. YqgS and LafA significantly contribute to the resistance of cell envelope-acting antimicrobials and unfavorable conditions. Notably, several studies have linked nisin and cephalosporin resistance in L. monocytogenes (18, 20, 25, 29). The identification of the genes related to cephalosporin resistance may promote the development of strategies to cope with the high resistance to these antibacterial agents (72).

L. monocytogenes colonizes food processing environments to form biofilms, which lead to biofilm-mediated antimicrobial resistance and stress tolerance, with negative implications for food safety (73–75). Biofilms could be responsible for persistent food contamination of bacteria, which in turn leads to the outbreak of *Listeria* infections (76, 77).The Δ*yqgS* and Δ*lafA* mutants displayed a greatly reduced capacity to form biofilm relative to wild-type strain. Bacterial adhesion, as a process indispensable to biofilm formation, is dictated by several factors, such as electrostatic and hydrophobic interactions, temperature, steric hindrance, and so on (78, 79). Given the negative charge of most bacteria and inert surfaces, electrostatic interactions are detrimental to the process of bacterial adhesion. The decline in biofilm forming capability of the Δ*yqgS* and Δ*lafA* mutants might be in part explained by the increased electrostatic repulsion.

LTA expression on group B streptococci plays an important role in human brain microvascular endothelial cells’ invasiveness (80), and LTA deficient mutant was less pathogenic than S. aureus wild-type (36). YqgS and LafA are crucial in efficient survival and replication of L. monocytogenes in macrophages. The mechanistic basis of the decreased pathogenic nature of the LTA mutation has not been resolved but might be connected to the alterations in envelope charge and envelope integrity, which increased sensitivity to antibacterial effectors produced by macrophages.

In conclusion, this study provided comprehensive identification of genes required for nisin resistance, which revealed novel mechanisms of nisin resistance in L. monocytogenes. LTA-related genes (*yqgS* and *lafA*) are required for biofilm formation, surface charge maintenance, and intracellular multiplication, which also significantly contribute to the resistance of cell envelope-acting antimicrobials and unfavorable conditions. These findings may provide novel targets for the development of food-grade inhibitors for nisin-resistant foodborne pathogens.

## MATERIALS AND METHODS

### Bacterial strains, plasmids, and growth conditions.

The L. monocytogenes strain EGDe (American Tissue Culture Collection) and the Escherichia coli strains EC1000 were used in this study. Unless otherwise noted, the bacteria were cultured in BHI (Hopebiol) at 37°C with shaking. Under necessary conditions, the concentrations of antibiotics used are as follows: gentamicin 25 μg/mL for L. monocytogenes and E. coli, chloramphenicol 5 μg/mL for L. monocytogenes, spectinomycin 100 μg/mL for L. monocytogenes and E. coli. All strains and plasmids used in this study are listed in Table S1.

### Construction of *mariner* transposon mutant library in L. monocytogenes.

To create a high-density transposon mutant library, the temperature-sensitive plasmid pGPA2 (81) was electroporated into L. monocytogenes. The mutant library was generated according to previously described methods (82). In brief, the plasmid-containing strains were incubated overnight in BHI with chloramphenicol at 30°C, and 200 μL of this culture was added into the BHI containing gentamicin (25 μg/mL) and nisin (25 ng/mL) for overnight culture. Then two successive passages were passed in BHI without antibiotics at 37°C. Subsequently, cultures were stored at −80°C with 50% (vol/vol) glycerol as mutant library stocks.

### Tn-seq analysis of genes involved in nisin resistance.

To identify genes that are involved in nisin resistance, 40 μL aliquots of the mutant library in L. monocytogenes were inoculated in BHI containing gentamicin (25 μg/mL) for overnight culture. Then three experimental replicates of approximately 2 × 10^7^ CFU were inoculated into 100 mL BHI or 100 mL BHI with 200 μg/mL nisin (Aladdin), which were incubated at 37°C for 16 h and then further processed for Tn-seq as described previously (83). Tn-seq sequencing was performed on Illumina Hiseq-PE150 (Personalbio), generating an average of 1G high clean data per sample.

Tn-seq data analysis was performed as previously described. In short, barcodes were split using the Galaxy platform and 16-nucleotide fragments of each read were mapped to the L. monocytogenes EGDe genome using Bowtie 2. The genome was subsequently divided in 25-bp windows and each alignment was sorted and indexed by IGV. Insertions were counted per window and then summed over the genes. Read counts per gene were adjusted to cover only the first 90% of the gene, because the final 10% of a gene were discarded as these insertions may not inactivate gene function. Subsequently, read counts were normalized to the total number of reads which mapped to the genome in each replicate, by calculating the normalized read-count reads per kilobase per million input reads (RKPM); RPTAM = (number of reads mapped to a gene × 10^6^)/(total mapped input reads in the sample × number of TA sites in this gene). The statistical analysis on the RPTAM values was performed by Cyber-T. Genes that significantly contributed to either nisin susceptibility or nisin resistance were determined when the Benjamini-Hochberg (BH) corrected *P*-value was < 0.05 and the difference in abundance of the transposon mutant during growth in BHI with or without nisin was > 5. Tn-seq data was provided in supplementary file 2.

### Construction of mutant strains.

Plasmid pWS3 was used to generate mutations through a process of double homologous recombination in L. monocytogenes, with a method based on the Cre-lox recombination system (82). The upstream and downstream homology arms of *yqgS* were PCR amplified from chromosomal DNA with *yqgS*_up_F/R and *yqgS*_down_F/R, respectively. The gentamicin maker was PCR amplified from pAT392 with pAT392_lox66_genta_F/R. These three DNA fragments were cloned in tandem (up-gm-down) on SmaI site of the pWS3 vector with NovoRec plus One step PCR Cloning Kit (novoprotein), resulting in plasmid pWS3- *yqgS* which used EC1000 as the cloning host. The recombinant plasmid with homologous arm and gentamicin maker was introduced in L. monocytogenes EGDe by electroporation. Positive clones were inoculated in prewarmed BHI medium without antibiotics and grown overnight at 37°C to get marked deletion mutants. To obtain the markerless mutants, the shuttle plasmid pWS3-erm-cre was electrotransformed into the marked mutants. Then cell culture was plated on BHI agar plates and incubated at 37°C to get the markerless mutations, verified by PCR and sequencing.

The same strategy was used to remove the *lafA*, *gtcA*, and *lmo1464* genes in L. monocytogenes to get the corresponding markerless mutations. Primers used in this study are listed in Table S1. All plasmid constructs and gene deletions were confirmed by DNA sequencing.

### Growth curves and nisin-mediated killing.

To draw growth curves of L. monocytogenes strains (wild-type and mutants), overnight cultures were diluted 100-fold in fresh BHI and BHI containing appropriate nisin or bacitracin. The absorbance of 630 nm (A630 nm) was measured every 1 h for 16 h. The same strategy was used to draw growth curves of L. monocytogenes wild-type, Δ*yqgS*, and Δ*lafA* mutants in unfavorable conditions.

Overnight cultures of L. monocytogenes strains were inoculated into the fresh BHI medium at a ratio of 1:100 and grew to exponential phase (OD600nm = 0.6 to 0.8), and then spun down and resuspended in phosphate buffer saline (PBS) to OD600nm = 0.65, next nisin (1,000 μg/mL) was added. Viable bacterial CFU counts were conducted after 2 h.

### Transmission electron microscopy and scanning electron microscopy.

The L. monocytogenes strains were grown to exponential phase (OD600nm = 0.8), followed by nisin (800 μg/mL) treatment in experimental groups for 1 h, after which the bacteria cultures were spun down and fixed with 2.5% glutaraldehyde in PBS for more than 4 h. The samples were washed three times in PBS, postfixed with 1% OsO4 for 1 h, and washed three times. Then the dehydration of samples used a graded series of ethanol (30%, 50%, 70%, 80%, 90%, 95%, 100%). The samples were dehydrated by alcohol for 20 min, and dehydrated in Hitachi Model HCP-2 critical point dryer. Later we can coat with gold-palladium in Hitachi Model E-1010 ion sputter for 4 to 5 min and observe in Hitachi Model SU-8010 SEM. The samples were transferred to absolute acetone for dehydration, placed in 1:1 mixture of absolute acetone and the final Spurr resin mixture, then transferred to 1:3 mixture of absolute acetone and the final resin mixture and to the final Spurr resin mixture for overnight. Specimens were heated at 70°C, then sectioned in LEICA EM UC7 ultratome, and sections were stained by uranyl acetate and alkaline lead citrate for 5 to 10 min respectively and observed in Hitachi Model H-7650 TEM.

### Determination of bacterial surface charge.

Bacterial surface charge was performed as described (84). Overnight cultures of L. monocytogenes strains were inoculated into the fresh BHI medium and grew to exponential phase (OD600nm = 0.6 to 0.8), then washed twice in 20 mM MOPS buffer (pH 7.0) and adjusted to OD600nm = 0.7. Bacterial aliquots were concentrated in half volume of 0.5 mg/mL equine cytochrome c dissolved in 20 mM MOPS buffer. After 10 min of incubation in the dark, the bacteria were centrifuged (14,000 rpm, 3 min) and the supernatant liquid was transferred to a 96-well plate. The absorbance was measured at 530 nm. The absorbance of samples only containing MOPS buffer was recorded as 100% binding, and the value of samples containing cytochrome c but lacking bacteria was recorded as 100% binding.

### Quantification of biofilm formation.

The overnight cultures were inoculated into the fresh BHI, adjusting the bacterial liquid concentration to 2 × 10^6^ CFU/mL. Then 200 μL of this bacterial liquid was absorbed into 96-well plate and incubated at 37°C for 48 h. The biofilm was quantified with the crystal violet (CV) assay (85). Briefly, culture was removed and washed with PBS three times to remove unattached cells. The wells were air-dried for 30 min, then 200 μL of methanol were added and set for 30 min. The wells were stained with 200 μL of 1% (wt/vol) crystal violet. After 30 min, rinse the well with 200 μL PBS for three times to remove excess staining and air dry the well. Finally, 200 μL glacial acetic acid (33%) was added to dissolve the crystalline violet bound to the biofilm. The absorbance of 595 nm was measured with the FLx800 Absorbance Reader (Biotech, USA). Experiments are performed at least in triplicate.

Fluorescence staining was used to observe the formation of biofilm under confocal microscope. 4 × 10^6^ CFU were added to 2 mL BHI with confocal dish and incubated at 37°C for 48 h. Cultures were discarded and dishes were washed thrice with PBS. Next, 200 μL SYTO9/PI dye solution was added to the culture dishes and treated for 30 min in the dark. After washing the dishes with PBS once, they were observed under a Confocal Laser Scanning Microscope LSM700.

### Antibiotic disk assays.

The antibiotic disk diffusion assays were performed as previously studied (18, 86). Briefly, the overnight cultures were diluted to OD600nm = 0.1 and swabbed onto BHI plates. Six-millimeter antibiotic disks were placed onto the corresponding BHI plates and incubated at 37°C for 24 h. The zones of inhibition were measured.

The size of inhibition zone of H_2_O_2_ was performed as mentioned above. It should be noted that 15 μL H_2_O_2_ (33%) was added to a 6-mL blank disk and left to dry for an hour. Subsequently, the H_2_O_2_ disks were placed onto the corresponding BHI plates and incubated at 37°C for 24 h to measure the size of inhibition zone.

### RAW264.7 macrophage infection.

Invasion and intracellular multiplication assays in RAW were performed as described (87). The L. monocytogenes strains were grown to exponential phase (OD600nm = 0.6 to 0.8), washed three times in dulbecco's modified eagle medium (DMEM), and diluted to MOI ≈25. Bacterial suspensions were transferred to macrophages and incubated for 1 h at 37°C, then washed three times and added fresh DMEM with gentamicin (25 μg/mL) for 1.5 h. Cells were lysed in 0.1% Triton X-100 and serially diluted for counting in BHI at different time points (0, 1, and 8 h).

### Statistical analyses.

To ensure the significance of the results, the data were analyzed using Student’s *t* test. The differences were considered statistically significant at the 95% level of confidence (*P* < 0.05).

### Data availability.

Illumina sequencing reads of L. monocytogenes in Tn-seq have been submitted in the NCBI server (Accession No. SRR17303189).
