# Supplementary material for: The Lipoteichoic Acid-Related Proteins YqgS and LafA Contribute to the Resistance of Listeria monocytogenes to Nisin
Source: Microbiol Spectr. 2022 Feb 23;10(1):e02095-21. doi: 10.1128/spectrum.02095-21 (PMC8865564; doi:10.1128/spectrum.02095-21)
Supplement: SUPPLEMENTAL FILE 2 — Supplemental material. Download SPECTRUM02095-21_Supp_1_seq4.pdf, PDF file, 0.3 MB [file spectrum02095-21_supp_1_seq4.pdf]

Table S1. Strains, plasmids, and PCR primers

Strain, primer, or plasmid	Relevant features (Primer sequence 5'-3')	Source or reference
Strains		
L. monocytogenes EGDe	Serotype 1/2a strain; genome is sequenced	(1)
E. coli EC1000	Cloning host strain	Invitrogen
Δ*yqgS*	EGDe Δ*yqgS*	This study
Δ*lafA*	EGDe Δ*lafA*	This study
Δ*virR*	EGDe Δ*virR*	Lab collection
Δ*gtcA*	EGDe Δ*gtcA*	This study
Δ*lmo1464*	EGDe Δ*lmo1464*	This study
Plasmids		
pGPA2	mariner delivery vector; Cm^r; Gm^r	(2)
pWS3	Temperature-sensitive integration vector; Spc^r	(3)
pWS3-erm-cre	Temperature-sensitive integration vector; Ery^r	This study
Primers		
pAT392_lox66_genta_F	TACCGTTCGTATAGCATACATTATACGAAGTTATGATAAACCCAGCGAACCATTTGAGG	(3)
pAT392_lox71_genta_R	TACCGTTCGTATAATGTATGCTATACGAAGTTATTCAATCTTTATAAGTCCTTTTATAA	(3)
genta2_F	ACCTCAAGTACCGTTCGTATAGCATACA	This study
genta2_R	GTGTAGCTTACCGTTCGTATAATGTATG	This study
Genta2_1_R	TCAAGGCAATCTGCCTCCTCA	This study
Genta2_2_F	ATGGAAAGACTAAATGCAACAACA	This study
pWS3_gm_ud_F1	GGGGATTTTATGCGTGAGAA	This study
pWS3_gm_ud_R1	GCTTCCAAGGAGCTAAAGAGG	This study
*yqgS*_up_F	AAGCTGAATTCCTGCAGCCCCGGGGATCAAAGTCATCGCA	This study
*yqgS*_up_R	TGTATGCTATACGAACGGTACTTGAGGTACGCTTTGACGCTAGCCGAA	This study
*yqgS*_down_F	GCATACATTATACGAACGGTAAGCTACACAGCAGGTCCATTCCAAGTGC	This study
*yqgS*_down_R	CTAGAACTAGTGGATCCCCCTCGCTGCAATCACGAAGGAA	This study
Check_*yqgS*_F	GAACCAGCTGAAGGTACGGT	This study
Check_*yqgS*_R	ACTGCAAGCCCGTATTCCAA	This study
*lafA*_up_F	AAGCTGAATTCCTGCAGCCCCCGAGCAAGTTCCTTCTCTGT	This study

lafA _up_R	TGTATGCTATACGAACGGTACTTGAGGT ACGTTCAAAGGAAAGAGAGGTC	This study
lafA _down_F	GCATACATTATACGAACGGTAAGCTACA CTGCGGACTGTAGGTATCCGT	This study
lafA _down_R	CTAGAACTAGTGGATCCCCCAAGTAAT CGGCCCTGGTGTG	This study
Check_*lafA*_F	GAAAATCCGCCTGCCCAAAC	This study
Check_*lafA*_R	CCGAACTTACAGGTGCTCCA	This study
*gtcA*_up_F	AAGCTGAATTCCTGCAGCCCCCAGCAA AAGCTGAGTGCG	
gtcA _up_R	TGTATGCTATACGAACGGTACTTGAGGT ATGGGCAAAAATTTGGACGAA	This study
gtcA _down_F	GCATACATTATACGAACGGTAAGCTACA CACCACGGAATCTTGTCTAACCAT	This study
gtcA _down_R	CTAGAACTAGTGGATCCCCCAAACCCC GGTTCTTCCAGAT	This study
Check_*gtcA*_F	TGCTTCCACTTGAAAACCTCCT	This study
Check_*gtcA*_R	ACAACATCCGCCAGAGCTAA	This study
*lmo1464*_up_F	AAGCTGAATTCCTGCAGCCCACCGTCG CTGTTTTTCCTGT	This study
*lmo1464*_up_R	TGTATGCTATACGAACGGTACTTGAGGT AGCGGTACTGTTAGCAGCAAT	This study
*lmo1464*_down_F	GCATACATTATACGAACGGTAAGCTACA CAAAAGCATGATGAAAAGATTCTGC	This study
*lmo1464*_down_R	CTAGAACTAGTGGATCCCCCAGGAGGG CAAAAGATGACGG	This study
Check_*lmo1464*_F	TGTGAATGCGCCTGGTAAAA	This study
Check_*lmo1464*_R	TCTTCCACAGAGCCAACCAT	This study

Table S2. *L. monocytogenes* genes involved in nisin resistance were determined by Tn-seq analysis.

**ORF**	**Gene**	**Product**	**Fold**	**BH**
**Cell wall envelope**				
lmo2477	*galE*	UDP-glucose 4-epimerase	5851.31	0.00E+00
lmo2554	*lafB*	galactosyltransferase	521.46	3.78E-10
lmo0644	*yqgS*	glycolipid synthesis protein	343.00	2.10E-10
lmo2555	*lafA*	N-acetylglucosaminyl-phosphatidylinositol biosynthesis protein	135.98	2.05E-09
lmo1695	*mprF*	hypothetical protein	10.92	2.13E-06
lmo2229	*pbp2*	penicillin-binding protein	6.69	8.51E-05
lmo0974	*dltA*	D-alanine--poly(phosphoribitol) ligase subunit 1	6.64	2.45E-02

lmo2553	*lafC*	hypothetical protein	6.54	2.70E-05
lmo0855	*ddl*	D-alanyl-alanine synthetase A	6.43	9.01E-05
lmo2505	*spl*	peptidoglycan lytic protein P45	6.18	2.66E-04
lmo2427	*ftsW*	cell division protein FtsW	5.31	1.04E-05
regulator				
lmo1745	*virR*	two-component response regulator	121.05	8.34E-09
lmo1022	*liaR*	two-component response regulator	77.68	1.61E-06
lmo0989		MarR family transcriptional regulator	44.69	1.22E-03
lmo1693	*recX*	recombination regulator RecX	22.93	9.51E-03
lmo1262		transcriptional regulator	11.09	6.85E-06
lmo0371		GntR family transcriptional regulator	10.93	7.36E-07
lmo0229		CtsR family transcriptional regulator	9.20	5.96E-04
lmo0651		transcriptional regulator	8.16	5.12E-04
lmo0031		LacI family transcription regulator	7.36	1.56E-05
lmo2493		ArsR family transcriptional regulator	6.61	7.09E-05
**ABC transporter**				
lmo2115	*anrB*	ABC transporter permease	157.10	2.10E-10
lmo2114	*anrA*	ABC transporter ATP-binding protein	131.39	4.47E-07
lmo1746	*virB*	ABC transporter permease	35.71	2.03E-06
lmo2768		hypothetical protein	21.54	1.40E-06
lmo2769		ABC transporter ATP-binding protein	13.72	4.47E-07
lmo1740		amino acid ABC transporter permease	13.62	7.17E-05
lmo2125		sugar ABC transporter substrate-binding protein	7.55	3.42E-05
**Phosphotransferase system (PTS)**				
lmo0916		PTS sugar transporter subunit IIA	16.50	4.85E-05
lmo0024		PTS mannose transporter subunit IID	7.49	1.97E-05
lmo2649	*ulaA*	PTS system ascorbate transporter subunit IIC	6.61	9.65E-06
lmo1971	*ulaA*	PTS ascorbate transporter subunit IIC	6.09	2.50E-02
lmo0021		PTS fructose transporter subunit IIA	5.52	7.17E-05
lmo0301		PTS beta-glucoside transporter subunit IIA	5.01	1.87E-03
**DNA repair**				
lmo1320	*polC*	DNA polymerase III PolC	6.47	3.73E-02
lmo1275	*topA*	DNA topoisomerase I	6.04	3.37E-03
lmo2523		single-strand DNA-binding protein	5.26	7.09E-05
**Secondary metabolites synthesis**				
lmo1809	*plsX*	glycerol-3-phosphate acyltransferase PlsX	390.97	3.78E-10
lmo2212	*hemE*	uroporphyrinogen decarboxylase	25.56	2.50E-04
lmo1557	*hemA*	glutamyl-tRNA reductase	24.91	7.28E-07
lmo2211	*hemH*	ferrochelatase	8.87	4.58E-02

lmo0884		protoporphyrinogen oxidase	7.57	2.03E-04
lmo1556	*hemC*	porphobilinogen deaminase	6.45	4.51E-03
lmo1932		heptaprenyl diphosphate synthase subunit I	6.01	7.39E-03
lmo2113		heme peroxidase	5.37	1.39E-03
**Oxidative phosphorylation**				
lmo2535	*atpB*	ATP synthase F0F1 subunit A	69.11	7.19E-08
lmo2531	*atpA*	ATP synthase F0F1 subunit alpha	44.08	4.13E-06
lmo2532	*atpH*	ATP synthase F0F1 subunit delta	20.30	4.03E-05
lmo2530	*atpG*	ATP synthase F0F1 subunit gamma	15.92	1.71E-05
lmo2529	*atpD*	ATP synthase F0F1 subunit beta	9.63	5.96E-05
**Others**				
lmo1229		hypothetical protein	415.25	6.85E-06
lmo2485		hypothetical protein	396.50	2.10E-10
lmo1249		hypothetical protein	191.15	1.14E-04
lmo1967	*telA*	toxic ion resistance protein	114.52	2.22E-08
lmo1841		hypothetical protein	93.01	1.02E-02
lmo1966		hypothetical protein	90.88	9.01E-07
lmo0290	*yycI*	hypothetical protein	63.11	4.16E-09
lmo2503		cardiolipin synthase	41.87	4.11E-08
lmo2811	*trmE*	tRNA modification GTPase TrmE	26.17	2.04E-03
lmo2156		hypothetical protein	25.13	9.20E-07
lmo0289	*yycH*	hypothetical protein	23.84	4.47E-07
lmo1757		hypothetical protein	20.46	9.40E-07
lmo1496	*greA*	transcription elongation factor GreA	18.95	2.73E-02
lmo2473		hypothetical protein	18.31	7.73E-07
lmo1289		internalin	14.77	3.08E-05
lmo0261		phospho-beta-glucosidase	14.59	2.27E-07
lmo2041	*mraW*	S-adenosyl-methyltransferase MraW	12.43	4.44E-05
lmo2077		glycoprotease	12.23	2.22E-02
lmo0167		hypothetical protein	10.81	1.32E-04
lmo2468	*clpP*	ATP-dependent Clp protease proteolytic subunit	10.53	2.48E-02
lmo1215		N-acetylmuramoyl-L-alanine amidase	10.22	2.97E-04
lmo2101		pyridoxal biosynthesis lyase PdxS	10.18	4.08E-02
lmo0052		hypothetical protein	9.45	2.86E-05
lmo1432		hypothetical protein	9.20	3.72E-02
lmo0737		hypothetical protein	8.70	5.98E-03
lmo0963	*htpX*	heat shock protein HtpX	8.04	7.28E-06
lmo1272	*rbgA*	ribosomal biogenesis GTPase	7.67	2.29E-04
lmo1888		hypothetical protein	7.34	1.14E-06
lmo0072		hypothetical protein	7.34	2.19E-04
lmo1842		hypothetical protein	7.00	6.48E-03

lmo1854		hypothetical protein	6.72	7.87E-06
lmo1484	*comEA*	competence protein ComEA	6.46	7.17E-04
lmo1820		serine/threonine protein kinase	6.33	8.79E-05
lmo0962	*lemA*	LemA protein	6.23	6.00E-05
lmo1598	*tyrS*	tyrosyl-tRNA synthetase	5.77	1.08E-02
lmo1800		protein-tyrosine phosphatase	5.77	1.01E-04
lmo0583	*azi, div*	preprotein translocase subunit SecA	5.69	6.54E-05
lmo0954		hypothetical protein	5.61	1.89E-04
lmo2484		hypothetical protein	5.48	7.28E-06
lmo2406		hypothetical protein	5.48	1.93E-04
lmo02333		hypothetical protein	5.40	1.14E-04

Table S3. *L. monocytogenes* genes related to nisin sensitivity as identified by Tn-seq analysis.

**ORF**	**Gene**	**Product**	**Fold**	**BH**
Lmo0215		hypothetical protein	0.15	7.28E-07
lmo0653		hypothetical protein	0.14	3.23E-05
lmo0955		hypothetical protein	0.11	3.51E-05
lmo1464		diacylglycerol kinase	0.06	6.85E-06
lmo2258		hypothetical protein	0.09	2.92E-07
lmo2380		monovalent cation/H+ antiporter subunit C	0.11	2.50E-02
lmo2549	*gtcA*	wall teichoic acid glycosylation protein GtcA	0.15	1.21E-05

1. Glaser P, Frangeul L, Buchrieser C, Rusniok C, Amend A, Baquero F, Berche P, Bloecker H, Brandt P, Chakraborty T, Charbit A, Chetouani F, Couve E, de Daruvar A, Dehoux P, Domann E, Dominguez-Bernal G, Duchaud E, Durant L, Dussurget O, Entian KD, Fsihi H, Garcia-del Portillo F, Garrido P, Gautier L, Goebel W, Gomez-Lopez N, Hain T, Hauf J, Jackson D, Jones LM, Kaerst U, Kreft J, Kuhn M, Kunst F, Kurapkat G, Madueno E, Maitournam A, Vicente JM, Ng E, Nedjari H, Nordsiek G, Novella S, de Pablos B, Perez-Diaz JC, Purcell R, Remmel B, Rose M, Schlueter T, Simoes N, et al. 2001. Comparative genomics of Listeria species. Science 294:849-52.
2. Zhang X, de Maat V, Guzman Prieto AM, Prajsnar TK, Bayjanov JR, de Been M, Rogers MRC, Bonten MJM, Mesnage S, Willems RJL, van Schaik W. 2017. RNA-seq and Tn-seq reveal fitness determinants of vancomycin-resistant *Enterococcus faecium* during growth in human serum. BMC Genomics 18:893.
3. Zhang X, Paganelli FL, Bierschenk D, Kuipers A, Bonten MJ, Willems RJ, van Schaik W. 2012. Genome-wide identification of ampicillin resistance determinants in *Enterococcus faecium*. PLoS Genet 8:e1002804.
